# Transplantation site influences the phenotypic differentiation of dopamine neurons in ventral mesencephalic grafts in Parkinsonian rats

**DOI:** 10.1016/j.expneurol.2017.01.010

**Published:** 2017-05

**Authors:** Marija Fjodorova, Eduardo M Torres, Stephen B Dunnett

**Affiliations:** Brain Repair Group, School of Biosciences, Cardiff University, Museum Avenue, Cardiff, Wales CF10 3AX, UK

**Keywords:** Parkinson's disease, Cell transplantation, Ventral mesencephalon, Striatum, Dopamine, A9 neuron, Girk2

## Abstract

Foetal midbrain progenitors have been shown to survive, give rise to different classes of dopamine neurons and integrate into the host brain alleviating Parkinsonian symptoms following transplantation in patients and animal models of the disease. Dopamine neuron subpopulations in the midbrain, namely A9 and A10, can be identified anatomically based on cell morphology and ascending axonal projections. G protein-gated inwardly rectifying potassium channel Girk2 and the calcium binding protein Calbindin are the two best available histochemical markers currently used to label (with some overlap) A9- and A10-like dopamine neuron subtypes, respectively, in tyrosine hydroxylase expressing neurons both in the midbrain and grafts. Both classes of dopamine neurons survive in grafts in the striatum and extend axonal projections to their normal dorsal and ventral striatal targets depending on phenotype. Nevertheless, grafts transplanted into the dorsal striatum, which is an A9 input nucleus, are enriched for dopamine neurons that express Girk2. It remains to be elucidated whether different transplantation sites favour the differential survival and/or development of concordant dopamine neuron subtypes within the grafts. Here we used rat foetal midbrain progenitors at two developmental stages corresponding to a peak in either A9 or A10 neurogenesis and examined their commitment to respective dopaminergic phenotypes by grafting cells into different forebrain regions that contain targets of either nigral A9 dopamine innervation (dorsal striatum), ventral tegmental area A10 dopamine innervation (nucleus accumbens and prefrontal cortex), or only sparse dopamine but rich noradrenaline innervation (hippocampus). We demonstrate that young (embryonic day, E12), but not older (E14), mesencephalic tissue and the transplant environment influence survival and functional integration of specific subtypes of dopamine neurons into the host brain. We also show that irrespective of donor age A9-like, Girk2-expressing neurons are more responsive to environmental cues in adopting a dopaminergic phenotype during differentiation post-grafting. These novel findings suggest that dopamine progenitors use targets of A9/A10 innervation in the transplantation site to complete maturation and the efficacy of foetal cell replacement therapy in patients may be improved by deriving midbrain tissue at earlier developmental stages than in current practice.

## Highlights

• Younger VM donor grafts are bigger and with enhanced A9-like dopamine neuron yield.
• A9:A10-like dopamine neuron ratios in VM grafts vary between transplantation sites.
• Dorsal striatum promotes A9-like dopamine neuron differentiation in VM grafts.
• Nucleus accumbens slightly enriches grafts with A10-like dopamine neurons.
• Transplantation site influences distribution of A9- and A10-like neurons in grafts.

## 1 Introduction

Parkinson's disease (PD) is a progressive neurodegenerative disorder characterised pathologically by a substantial loss of melanised dopamine neurons in the substantia nigra pars compacta (SNpc) and a subsequent failure to supply dopamine to the putamen, which results in impaired motor, cognitive and neuropsychiatric functions in patients. There is no therapy available to cure PD and current pharmacological treatments, such as levodopa, only address the symptoms by restoring dopamine transmission in the striatum, alleviating some motor deficits but often having little effect, or even impairing, cognitive functions (Hernandez et al., 2014, Obeso et al., 2011, Schneider et al., 2013). In contrast, a series of proof-of-principle clinical trials demonstrated that human foetal ventral mesencephalic (VM) dopamine progenitors, ectopically transplanted into the putamen and in some cases also into either the caudate nucleus or the SNpc, resulted in significant long-term clinical improvement of motor function in patients and in several cases there was no further need for levodopa treatment (Freed et al., 1992, Hagell et al., 1999, Kefalopoulou et al., 2014, Lindvall et al., 1992, Lopez-Lozano et al., 1997, Mendez et al., 2002, Mendez et al., 2005, Mendez et al., 2008, Wenning et al., 1997). We have recently demonstrated that human VM transplants are also capable of ameliorating non-motor dysfunctions in a rat model of PD (Lelos et al., 2016).

The subtypes of dopamine neurons within embryonic VM grafts have been the focus of many studies. Based on anatomical morphology from histochemical descriptions, out of nine dopamine cell groups in the brain three are present in the developing mammalian midbrain – A9 neurons of the SNpc, A10 neurons of the ventral tegmental area (VTA), and A8 neurons of the retrorubral field (Dahlstrom and Fuxe, 1964). Anatomically the key sites are the SNpc and VTA collectively containing ≈ 90% of all dopamine neurons in the rat midbrain (German and Manaye, 1993, Nair-Roberts et al., 2008). In a very simplified model, A9 neurons and their ascending axonal projections to the dorsal striatum (dSTR) in rodents (caudate nucleus and putamen in primates) comprise the nigrostriatal pathway while A10 neurons in the mesocorticolimbic pathway extend their axons primarily to the ventral striatum, also known as nucleus accumbens (N.Acc) and cortical areas, but also to the olfactory tubercle, septum and amygdala (reviewed in Björklund and Dunnett, 2007).

Subsequently, A9 and A10 neurons in the midbrain can be identified based on cell morphology and expression profiles. Tyrosine hydroxylase-immunoreactive (TH-ir) A9 neurons located in the ventral tier of the SNpc as well as the ventrolateral region of the VTA are large and angular in shape and most co-express G protein-gated inwardly rectifying potassium channel 2 (Girk2), while TH-ir A10 neurons located in the VTA and dorsal tier of the SNpc are smaller and round in shape and mostly co-express the calcium binding protein Calbindin (Reyes et al., 2012, Thompson et al., 2005). However, authors also reported that strong levels of Girk2 protein can be detected in up to 5–25% of Calbindin-ir/TH-ir neurons in the dorsal tier of the SNpc in mice and humans. Nevertheless, several studies have demonstrated that these distinctive morphological features of the two dopamine neuron populations are retained after transplantation of embryonic VM and Girk2 and Calbindin currently are best available surrogate markers that can be used to define A9- and A10-like neurons once their positional criteria are lost within grafts (Bye et al., 2012, Grealish et al., 2010, Mendez et al., 2005, Thompson et al., 2005).

Increasing lines of evidence indicate that the restoration of function by dopamine grafts is mainly due to the presence of A9-like neurons (Grealish et al., 2010, Kuan et al., 2007). Although both subpopulations of dopamine neurons are present in VM grafts in the dSTR, these grafts are enriched for the A9-like neurons (Bye et al., 2012, Somaa et al., 2015). The use of younger donor tissue and presence of meningeal cells overlying the VM in graft preparation have already been identified among the factors that favour the survival of A9-like neuron population in grafts in dSTR and result in a more effective cell replacement therapy in animal models of PD (Bye et al., 2012, Somaa et al., 2015, Torres et al., 2008, Torres et al., 2007). We set out to determine whether other variables, such as the host environment external to the graft, may have influenced this improved yield of functionally important A9-like neurons in transplants.

In particular, we questioned whether targets of A9 innervation in the dSTR might have contributed to these effects. To this extent, we investigated the commitment of dopamine neuron progenitors to the A9- and A10-like fates by grafting younger, embryonic day 12 (E12), and conventional age (E14) rat VM tissue into cerebral regions containing targets of either A9, A10 or noradrenaline innervation. We examined whether different transplantation sites favour the differential survival and/or development of concordant dopamine neuron subtypes within the grafts. We believe this to be the first systematic comparison of A9-/A10-like cell populations in rat VM grafts transplanted at different developmental stages into various environments in the context of PD.

## 2 Methods

### 2.1 Subjects

Sprague Dawley rats (Charles River, UK) were housed under standard conditions on a 14 h:10 h light/dark cycle with ad libitum access to food and water. All experiments were conducted under UK Home Office personal and project licences in accordance with the requirements of the UK Animals (Scientific Procedures) Act 1986 and with the approval of the local Cardiff University Ethics Review Committee. Every effort was made to minimise the number of animals used and their suffering.

### 2.2 Experimental design

To test our hypothesis, female rats were unilaterally lesioned with the selective catecholamine neurotoxin 6-hydroxydopamine (6-OHDA) into the nigrostriatal pathway and the lesions confirmed with amphetamine-induced rotations at 4 weeks post-lesion. Prior to transplantation, rats were separated into 8 balanced groups based on rotation performance [see Table 1; Group, F_(7,28)_ = 0.03, n.s.]. All transplants were made on the dopamine-depleted side of the brain. To reduce the number of animals used in the experiment each animal received 2 grafts implanted into widely separated sites in the denervated hemisphere, with the first transplant into either dSTR or N.Acc and the second transplant into either prefrontal cortex (PFC) or hippocampus (HPC). Efficacy of the grafts was confirmed with amphetamine-induced rotation tests at 4 and 6 weeks post-graft. Following the final rotation test animals were perfused and brain tissue taken for histological analysis.

### 2.3 Medial forebrain bundle lesions

All surgeries were performed in a Kopf stereotaxic frame where anaesthesia was maintained at 2–3% Isoflurane (AbbVie Ltd., Maidenhead, UK) in a 2:1 mixture of oxygen and nitrous oxide. For the unilateral nigrostriatal lesion, rats received an intra-cerebral injection of 6-OHDA neurotoxin into the right medial forebrain bundle following a recently refined protocol (Torres et al., 2011). Each animal was injected with 12 μg (free-base) of 6-OHDA hydrobromide (Sigma-Aldrich, Gillingham, UK) in 3 μl of 0.025% ascorbate saline at the following stereotaxic coordinates: − 4.0 mm caudal from bregma (AP); − 1.3 mm lateral from the midline (ML); − 7.0 mm deep from dura (DV).

### 2.4 Procurement of embryonic ventral mesencephalic cells

Pregnant dams were bred in-house following a previously established protocol (Weyrauch et al., 2009). Briefly, females in oestrus were established by the vaginal lavage method on the morning of breeding day. Eligible females were paired with a male at 1:1 ratio in the male's home cage for a maximum of 3 h, typically between 10:30 a.m. and 1:30 p.m. Females then returned to their home cages and the day of mating was recorded as E0. At 10 a.m. on the morning of E12, rats were lightly anaesthetised in an induction chamber with 2–3% Isoflurane in oxygen and the pregnancy was confirmed if palpation of the abdomen revealed several swellings in the uterine horns. Embryos were harvested straight away if the desired embryonic age was E12, alternatively pregnant dams returned to their home cages until E14. The short mating-period protocol used here produced embryos with a crown-rump length (CRL) of 6.1 ± 0.3 mm at E12 and 11.2 ± 0.4 mm at E14 (CRL reported as mean ± standard deviation).

The VM tissue was harvested from three E12 and two E14 litters and dissected from each embryo according to a standard protocol (Dunnett and Björklund, 1992). VMs from all donors in the same litter were pooled together and quasi-singe cell suspensions were prepared as previously described (Torres et al., 2007). The viability for each sample was determined to be > 95% using Trypan Blue exclusion criteria. For transplantation, the VM cells were re-suspended in a sufficient volume of Dulbecco's minimum Eagle Medium F-12 (Gibco, Paisley, UK) containing 0.05% DNAse (deoxyribonuclease I from bovine pancreas, Sigma-Aldrich) to obtain the required concentration of 1/2 VM per 2 μl and stored on ice during surgery.

### 2.5 Transplantation

Six weeks post-lesion, each animal was transplanted with the number of cells equivalent to 1/2 VM, rather than with a fixed number of cells, to allow a comparison to be made between grafts of similar potential at the different donor ages. Thus, each rat received grafts into 2 designated sites, each graft containing approximately 55,000 cells in E12 group or 250,000 cells in E14 group. Overall, a total of 9 animals received grafts of each donor age transplanted into each cerebral target. Stereotaxic co-ordinates were as follows: **dSTR** AP: + 0.6 mm, ML: − 3.0 mm, DV: − 5.0 & − 4.5 mm; **N.Acc** AP: + 1.6 mm, ML: − 1.5 mm, DV: − 7.5 & − 7 mm; **PFC** AP: + 4.7 mm, ML: − 2.0 mm, DV: − 1.9 & − 1.4 mm; **HPC** AP: − 5.2 mm, ML: − 5.2 mm, DV: − 5.4 & − 4.9 mm (Paxinos and Watson, 2003). Cells were transplanted in a single track at 2 depths 0.5 mm apart. The cell suspension was injected into the brain at a rate of 1 μl/min over 2 min using the same set up as for the 6-OHDA lesion surgery. After the first minute, the cannula was raised 0.5 mm and the second half of cells was injected at the new depth. The cannula was left in place for 3 min after the injection before being withdrawn to prevent drawback of the cells up the needle track.

### 2.6 Behavioural testing

The extent of dopamine depletion in the striatum was evaluated 4 weeks post-lesion based on the drug-induced rotational behaviour. Amphetamine (Methamphetamine hydrochloride, Sigma-Aldrich) was dissolved in sterile saline at 2.5 mg/ml and administered via i.p. injection at a dose of 1 ml/kg, immediately prior to testing. The animals were placed in 30 cm diameter round bottomed bowls housed in 50 cm high perspex cylinders and harnessed to an automated rotometer system (Rotomax System, AccuScan Instruments Inc., Columbus, USA) following a previously established protocol (Ungerstedt and Arbuthnott, 1970). Grafted rats were tested 4 and 6 weeks post-transplantation using the same method. Drug-induced rotation tests were performed blind to the treatment of the animals. The collected data are reported as the net number of rotations (ipsilateral minus contralateral) over the full 90 min session.

### 2.7 Perfusion

Six weeks post-graft, rats were terminally anaesthetised with an overdose of sodium pentobarbital (Euthatal, Merial, UK) and transcardially perfused with 0.1 M phosphate buffered saline (PBS) followed by 1.5% w/v paraformaldehyde (Fisher Scientific, Loughborough, UK) in 0.1 M PBS. The brains were post-fixed in the same fixative solution overnight before being transferred into a solution of 25% sucrose in PBS. After equilibration in the sucrose solution, 40 μm thick coronal sections through the brain were cut on a Leica freezing stage sledge microtome and collected in 1:6 series.

### 2.8 Immunohistochemistry

All bright-field and fluorescent immunohistochemistry (IHC) was performed on free floating sections on an automated shaker. The tissue was first incubated for 1 h in a blocking solution of 3% normal horse serum (NHS, Gibco) before being bathed in primary antibody with 1% NHS overnight at room temperature. The next day, the sections were incubated in secondary antibody (1:200) solution with 1% NHS for 3 h, washed, and then immersed in Vectastain Elite ABC (Vector Laboratories Ltd., Peterborough, UK) solution for 2 h prior to being stained with DAB (diaminobenzidine tetrahydrochloride hydrate, Sigma-Aldrich). The primary antibodies and dilution factors used in either bright-field or fluorescent IHC were as follows: mouse anti-NeuN (1:1000; Millipore, Feltham, UK), rabbit anti-TH (1:2000; Millipore), mouse anti-TH (1:1000; Millipore), rabbit anti-Girk2 (1:250; Alomone Labs, Jerusalem, Israel), and mouse anti-Calbindin (1:10,000; Sigma-Aldrich). All Alexa fluorescent secondary antibodies were used at 1:200 dilution.

### 2.9 Quantification and microscopy

All quantifications of cell populations in the grafts were performed blind to the experimental condition. Neuron quantification in the grafts from NeuN stained sections was carried out on an automated stereology microscope (Olympus BX50) at × 100 magnification using image analysis C.A.S.T. – grid software version 1.6 (Olympus, Ballerup, Denmark). The area of the graft was outlined by the user and the software set up randomised statistical sampling within the defined area so that at 150–200 cells per animal were counted from at least 200 sampling fields. Sampling parameters, i.e. area of the sampling frame (285 μm^2^) and step size between the samples, were held constant for all animals in each experiment. Total cell counts in the grafts for each staining were estimated using the Abercrombie correction formula (Abercrombie, 1946). To assess the extent of 6-OHDA lesions, numbers of TH-ir cells were counted at × 10 magnification on the side ipsilateral and contralateral to the lesion in one midbrain section at the level of the medial terminal nucleus of the accessory nucleus of the optic tract as an anatomical landmark to distinguish cells in the VTA and SNpc (Dowd and Dunnett, 2004).

To quantify cells labelled with fluorochromes, pictures of all sections containing the graft were taken on a Zeiss Image Z2 microscope equipped with the imaging system ApoTome (Oberkochen, Germany) to generate deblurred optical sections of fluorescence samples at × 10 magnification and single- and double-labelled cells were counted in Adobe Photoshop CS5 software (Adobe Systems Inc., California, USA). Using a grid generated in Photoshop as a reference, constant-size sampling field was selected from the widest area of the graft and the distribution of Girk2-ir/TH-ir and Calbindin-ir/TH-ir neurons within the graft was analysed by counting cells in the fields sampled from either the periphery (25% width of the graft from each side) or the centre (50%) of the graft and grouping them accordingly for statistical analysis.

### 2.10 Statistical analysis

All data are expressed as group mean ± standard error of the mean (SEM). Statistical analyses were performed in SPSS Statistics 23 (IBM Corp., Armonk, USA). A general linear model (GLM) univariate function was used to perform two- and higher factor analyses of variance (ANOVAs) to analyse data from IHC staining. A four-way ANOVA with repeated measures was used to analyse behavioural data from drug-induced rotation tests. This analysis was performed orthogonally, treating grafts in the dSTR or N.Acc as the primary graft and grafts in the PFC or HPC as the secondary graft, to investigate whether PFC or HPC subgroups differed in either dSTR or N.Acc groups. Bonferroni correction for multiple comparisons was used to reveal significant differences between individual groups and results were considered to be significant if p < 0.05 (*), with higher levels of significance (**p < 0.01, ***p < 0.001) also indicated where appropriate.

One grafted rat was transferred from E14 N.Acc group to E14 dSTR group for all analyses after examination of TH-stained brain sections revealed that the first graft was positioned in the dSTR instead of N.Acc. Furthermore, 3 of 18 animals in the PFC and 1 of 18 in the HPC groups were missing grafts. This was most likely because cells were injected into either a subdural or a sub-ventricular space as no trace of cells was found in the target or adjacent brain regions. The resulting numbers of grafts in each group used in further analyses were as follows: dSTR – 9 E12 and 10 E14 grafts, N.Acc – 9 E12 and 8 E14 grafts, PFC – 8 E12 and 7 E14 grafts, HPC – 9 E12 and 8 E14 grafts.

## 3 Results

### 3.1 Functionality of the grafts

**Fig. 1:**
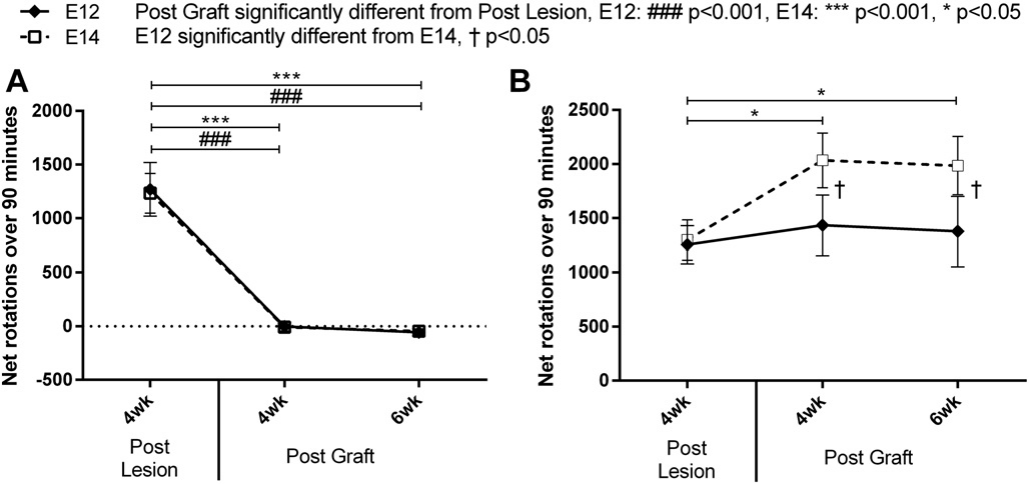
VM grafts affect drug-induced rotational bias in the dSTR (A) and N.Acc (B) groups. Mean (ipsilateral minus contralateral) rotation score over 90 min following an i.p. injection of 2.5 mg/kg metamphetamine in lesioned and grafted rats in the E12 and E14 groups. Data are collapsed across the secondary graft factor. (A) Both donor age graft groups produced a recovery of the lesion-induced behavioural deficit in the dSTR graft group. There was a significant change from ipsilateral rotation observed post-lesion to a net contralateral rotation post-graft, the classic over-compensatory response. (B) Animals in the E14 N.Acc graft group exhibited enhanced (*) ipsilateral rotational behaviour post-graft. Error bars indicate ± SEM, significance levels: * or †p < 0.05, *** or ###p < 0.001.

Rotational asymmetry in response to the indirect dopamine agonist, amphetamine, was assessed at 4 weeks post-lesion and at 4 and 6 weeks post-graft. Post-lesion, all animals exhibited a rotational bias toward the lesioned side, a classic behavioural deficit induced by the 6-OHDA lesion to the nigrostriatal pathway (see Fig. 1). Grafts in the dSTR and N.Acc produced significantly different drug-induced rotational behaviours in animals [Primary graft, F_(1,28)_ = 55.57, p < 0.001]. All rats, which had received grafts into the dSTR, showed a significant behavioural recovery [Fig. 1A; Lesion vs dSTR at 4 and 6 week., both p < 0.001] and some now rotated contralaterally to the lesioned side, a classic ‘overcompensation’ response (Dunnett et al., 1981), while grafts in the N.Acc exacerbated the rotational bias toward the lesioned side [Fig. 1B; Lesion vs N.Acc at 4 and 6 week., both p < 0.05]. Grafts in either the PFC or HPC did not interfere with drug-induced rotational response provided by grafts in either dSTR or N.Acc [secondary graft, main effect, F_(1,28)_ = 1.31, n.s. and no significant interactions]. Therefore, behavioural data presented in Fig. 1 are collapsed across the secondary graft factor.

Rotational behaviour between the E12 and E14 groups was very similar in the dSTR group. In both donor age groups, grafts in the dSTR induced an equally high significant behavioural recovery [Fig. 1A; Lesion vs E12 and E14 dSTR at 4 and 6 week., all p < 0.001]. On the other hand, post-graft rotational behaviour in the N.Acc graft animals was significantly different between the two donor age groups at both 4 and 6 week time points [Fig. 1B; E12 N.Acc vs E14 N.Acc at 4 and 6 week., both p < 0.05]. Thus, rotational bias post-graft was significantly higher than post-lesion in the E14 N.Acc [Fig. 1B; Lesion vs E14 N.Acc at 4 and 6 week., both p < 0.05], whereas the graft did not affect rotational asymmetry in the E12 N.Acc group [Fig. 1B; Lesion vs E12 N.Acc at 4 and 6 week., both n.s.].

### 3.2 Transplantation site does not influence total neuron yield in ventral mesencephalic grafts

**Fig. 2:**
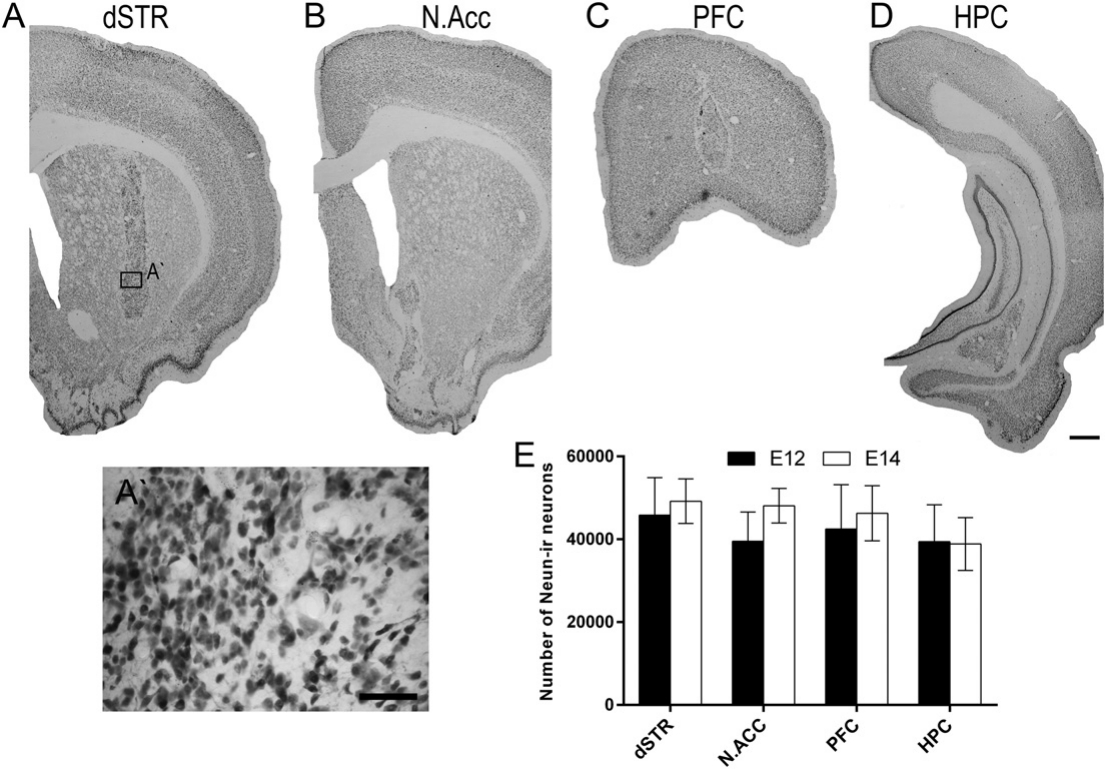
Transplantation site does not influence total neuron yield in VM grafts in the Parkinsonian brain. (A–D) Photomicrographs illustrating NeuN staining of E12 VM grafts in different transplantation targets: (A) dSTR, (B) N.Acc, (C) PFC, and (D) HPC. There is a clear boundary between the graft and the surrounding host brain. Neurons are homogeneously distributed within the graft and their density appears to be higher in the graft than in the surrounding brain tissue. (A′) High magnification image from (A), illustrating the morphology of the NeuN-ir cells within the graft. (E) Quantification of NeuN-ir cells at 6 weeks post-graft revealed no significant differences between the donor age groups and transplantation site groups (n.s.). Columns depict group means; error bars illustrate ± SEM. Scale: A–D, 500 μm; A′: 50 μm. Abbreviations: dorsal striatum (dSTR), nucleus accumbens (N.Acc), prefrontal cortex (PFC), hippocampus (HPC).

Examination of NeuN-stained sections revealed surviving grafts in all transplantation sites with large numbers of neurons homogeneously distributed within the grafts as depicted in Fig. 2A–D. On average, there were over 40,000 NeuN-ir cells in each graft. Given that E14 VMs contained almost 5 times more cells than E12 VMs at the time of transplantation, the yield of NeuN-ir neurons might have been expected to be different between the two donor age groups. However, as shown in Fig. 2E, the yield of grafted neurons was very similar in all groups and was not affected by the donor age [F_(1,59)_ = 0.51, n.s.] or the transplantation site [F_(3,59)_ = 0.45, n.s.].

### 3.3 Younger donor tissue and striatal environment enhance dopamine neuron survival and integration of ventral mesencephalic grafts

**Fig. 3:**
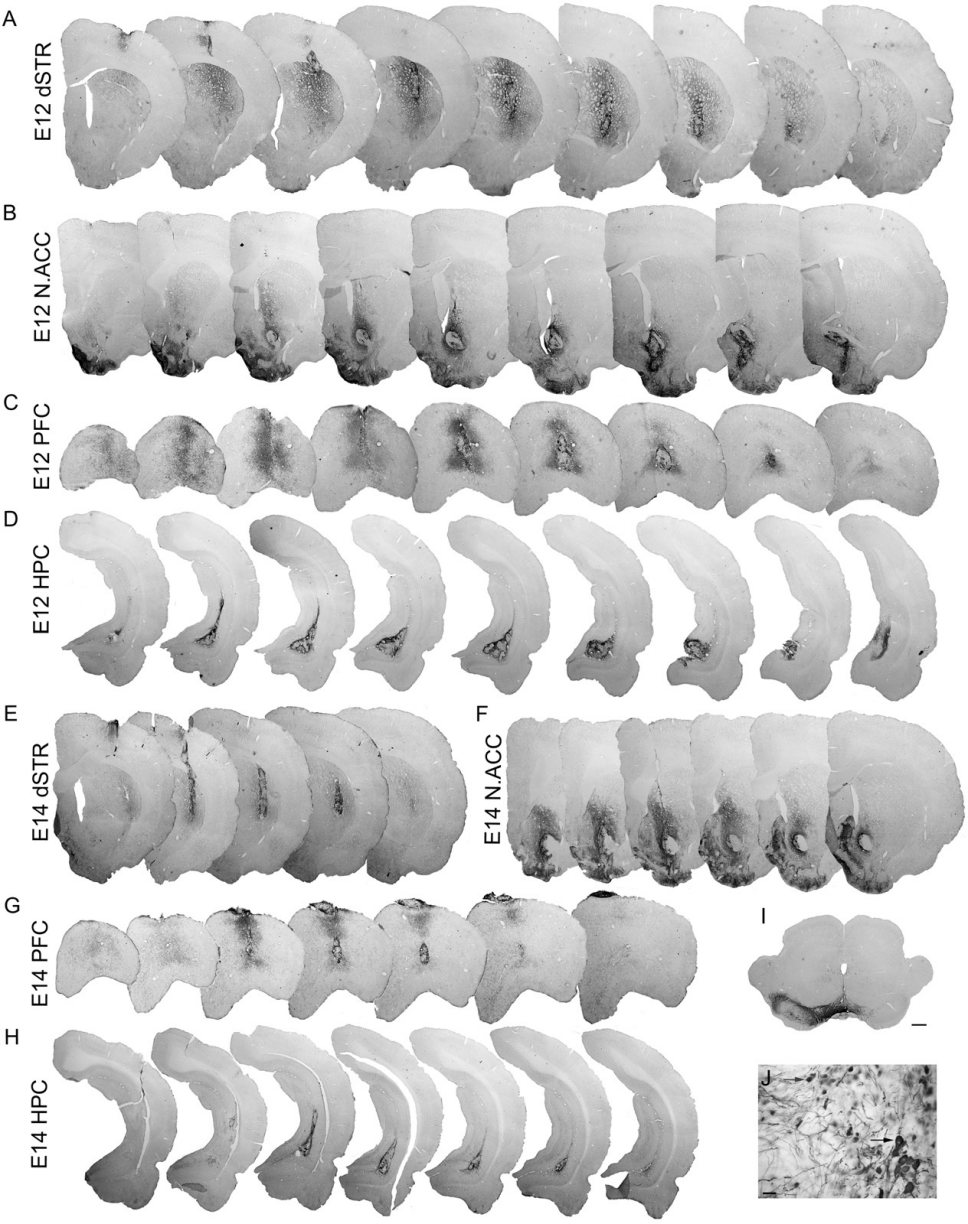
Immunohistochemical analysis of dopamine neuron survival in VM grafts in a rat model of PD. Photomicrographs of TH staining in E12 VM grafts in the dSTR (A), N.Acc (B), PFC (C), and HPC (D), and E14 VM grafts in the same cerebral targets (E, F, G, and H, respectively) at 6 weeks post-transplantation illustrating abundant dopamine neuron survival and integration into the host brain. (I) Photomicrograph of TH staining in a coronal section through 6-OHDA lesioned midbrain demonstrating extensive ablation of the host dopaminergic midbrain pathways. (J) High magnification image illustrating the morphology of TH-ir neurons within the transplant and graft-derived dopaminergic axons extended into the surrounding tissue. Grafts contained larger elongated TH-ir neurons suggestive of an A9-like phenotype (black arrow head) as well as smaller spherical neurons indicative of A10-like phenotype (grey arrow head). Scale bars: 500 μm (A–I) and 20 μm (J). Abbreviations: dorsal striatum (dSTR), nucleus accumbens (N.Acc), prefrontal cortex (PFC), hippocampus (HPC).

At 6 weeks post-transplantation, VM graft survival and integration into 6-OHDA lesioned rat brain was examined by staining for TH (Fig. 3). Extensive ablation of the host dopaminergic midbrain pathways was evidenced by virtually no (consistently < 5%) surviving TH-ir neurons in the lesioned SNpc (the origin of the A9 dopamine neurons) and a partial (≈ 35–50%) loss of TH-ir neurons in the VTA (the origin of the A10 dopamine neurons; Fig. 3I). TH staining confirmed surviving dopamine-rich grafts in all transplantation sites, and graft-derived TH-ir fibres were observed innervating the host tissue in all groups (Fig. 3A–H). Dopaminergic innervation arising from E12 VM grafts appeared denser and more extensive in all cerebral targets compared to E14 VM grafts although outgrowth was not quantified. As clearly visible in Fig. 3, in both donor age groups, grafts in the dSTR and N.Acc showed the greatest level of innervation of the host tissue, with substantial fibre outgrowth also seen in the PFC. In the HPC, dopamine neurons survived in abundance but exhibited only very sparse axonal growth and only into the immediate vicinity of the transplant site. E14 grafts contained most TH-ir neurons in the periphery of the graft and only a few in the centre of the graft (Fig. 3E–H). E12 grafts displayed a more homogeneous distribution of cells and a high density of TH processes within the graft (Fig. 3A–D).

**Fig. 4:**
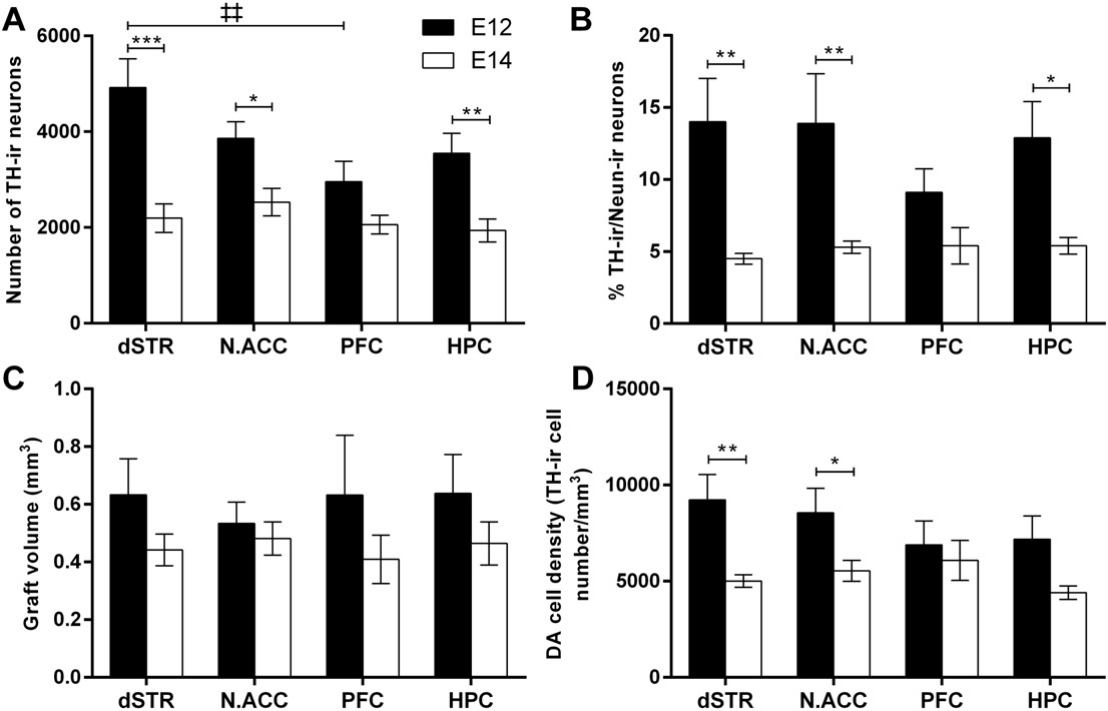
Donor tissue age and transplantation site influence the number and density of TH-ir neurons in 6-week-old VM grafts in a rat model of PD. Data portray the total number of TH-ir neurons (A) and the percentage of TH-ir/NeuN-ir cells (B) in the grafts, graft volumes (C) and the density of TH-ir neurons in the grafts (D). Columns depict group means; error bars illustrate ± SEM; significance levels: *p < 0.05, ** or ‡‡p < 0.01, ***p < 0.001. Abbreviations: dorsal striatum (dSTR), nucleus accumbens (N.Acc), prefrontal cortex (PFC), hippocampus (HPC).

Younger donor tissue resulted in significantly more TH-ir cells within the grafts in most locations [Fig. 4A; Donor Age, F_(1,60)_ = 36.18, p < 0.001]. E12 grafts produced a significantly higher yield of TH-ir cells than E14 transplants in the dSTR [p < 0.001], N.Acc [p < 0.05] and HPC [p < 0.01] but not the PFC. Interestingly, in the E12 donor age group grafts in the dSTR were the most populous in TH-ir cell numbers in comparison to grafts in any other location, whereas for the E14 donor age group the largest grafts were in the N.Acc. Also, grafts in the HPC contained unexpectedly high numbers of TH-ir cells, especially in the E12 donor age group. However, only E12 grafts in the dSTR produced a significantly higher yield of dopamine neurons than in the PFC [Transplantation Site, F_(3,60)_ = 2.98, p < 0.05; E12 dSTR vs E12 PFC, p < 0.01, although this might be an artefact as some of the very anterior sections of the PFC containing the graft were lost during tissue acquisition].

Given that the total number of surviving neurons was not significantly different between the groups, we should expect a higher proportion of TH-ir/NeuN-ir cells in the E12 donor age group than E14 group to account for the significantly higher dopamine neuron yield in E12 grafts. Indeed, E12 grafts were more enriched for dopamine neurons than E14 grafts in most transplantation sites [Fig. 4B; Donor Age, F_(1,59)_ = 24.02, p < 0.001]. In line with significant differences in the number of TH-ir neurons, grafts derived from E12 rat embryos yielded a higher percentage of TH-ir/NeuN-ir neurons than E14 grafts in the dSTR [p < 0.01], N.Acc [p < 0.01] and the HPC [p < 0.05] but not the PFC.

Generally, E12 grafts were slightly but significantly bigger in volume than E14 grafts in all transplantation sites [Fig. 4C; Donor Age, F_(1,60)_ = 4.12, p < 0.05], despite the smaller numbers of cells implanted, whereas no significant differences were found between individual groups in graft volume. Despite the fact that brain tissue in each transplantation site is different in cell composition and density it did not affect the growth of grafts [Transplantation Site, F_(3,60)_ = 0.06, n.s.; Donor Age × Transplantation Site, F_(3,60)_ = 0.22, n.s.]. Analysis of dopamine neuron densities revealed that E12 grafts were significantly denser than E14 grafts [Fig. 4D; Donor Age, F_(1,60)_ = 14.30, p < 0.001]. TH-ir cell density was significantly higher in E12 grafts than E14 grafts in the dSTR [p < 0.01] and N.Acc [p < 0.05]. The density of TH-ir neurons in the grafts varied little between transplantation sites [Transplantation Site, F_(3,60)_ = 0.76, n.s.].

### 3.4 Transplantation site influences the phenotypic differentiation of dopamine neurons in ventral mesencephalic grafts

**Fig. 5:**
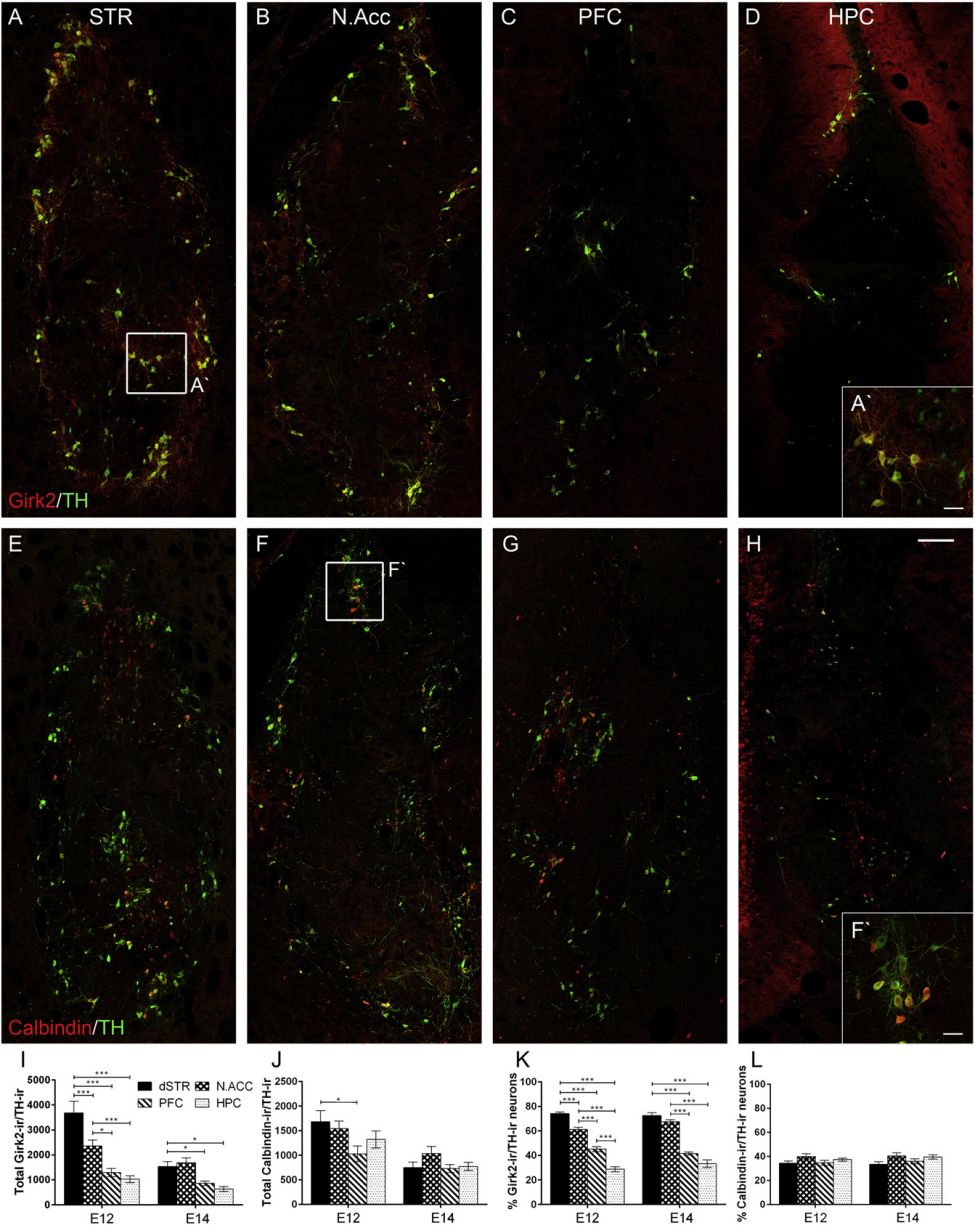
Transplantation site influences A9-like dopamine neuron specification in VM grafts. Coronal sections through E12 VM grafts illustrating TH-ir (green) neurons co-expressing Girk2 (red: A–D) or Calbindin (red: E–H) in grafts in the dSTR (A, E), N.Acc (B, F), PFC (C, G) and HPC (D, H). Note the increase in the Girk2-ir/TH-ir neuron population in grafts in the dSTR compared to other grafts. (A′ and F′) High magnification images from (A and F), illustrating the co-localisation of Girk2 (A′) and Calbindin (F′) with TH and morphology of double labelled neurons within the transplant. (I) Total number of Girk2-ir/TH-ir neurons and (J) Calbindin-ir/TH-ir neurons within the grafts at each donor age and transplantation site. (K) Quantification of the proportion of Girk2-ir/TH-ir neurons and (L) Calbindin-ir/TH-ir neurons out of total TH-ir neurons within the grafts. The presence of targeted midbrain innervation of the transplantation site significantly increased the number and proportion of A9-like neurons in the grafts. Scale bars: 100 μm (A–H) and 25 μm (A′ and F′). Columns depict group means; error bars illustrate ± SEM; significance levels: *p < 0.05, ***p < 0.001. Abbreviations: dorsal striatum (dSTR), nucleus accumbens (N.Acc), prefrontal cortex (PFC), hippocampus (HPC).

Fluorescent double staining for Girk2/TH and Calbindin/TH enabled visualisation of the Girk2-ir A9-like and Calbindin-ir A10-like dopamine neurons in the grafts and assessment of the effects of differential presence of A9 and A10 innervation targets in the transplantation sites on the phenotypic differentiation of transplanted dopamine progenitors (Fig. 5). Examination of labelled sections revealed presence of A9- and A10-like dopamine neurons in all grafts where Girk2 and Calbindin co-localised with TH in the cytoplasm of grafted neurons (Fig. 5A′,F′). The majority of A9-like neurons were large and elongated in shape, while A10-like neurons were smaller and more spherical. The yield of A9-like dopamine neurons was predicted to be higher in the dSTR and A10-like dopamine neuron yield was expected to be higher in the N.Acc and PFC due to differential presence of A9 and A10 innervation targets in these brain regions. Indeed, while Girk2-ir/TH-ir cells were abundant in grafts in the dSTR and N.Acc in both donor age groups, there were noticeably fewer Girk2/TH double labelled cells in grafts in the PFC and HPC as depicted in Fig. 5A–D. Also, Girk2-ir fibre density appeared to be higher in the immediate vicinity of grafts in the dSTR as compared to grafts in other transplantation sites. However, differences in the populations of Calbindin/TH double labelled cells between transplantation sites were less prominent (Fig. 5E–H).

There was a highly significant difference between A9- and A10-like dopamine neuron populations in the grafts [within-subject factor – Staining, F_(1,60)_ = 111.48, p < 0.001] influenced by both the host environment [Transplantation Site, F_(3,60)_ = 14.19, p < 0.001] and the VM age [Donor Age, F_(1,60)_ = 31.59, p < 0.001; Transplantation Site × Donor Age, F_(3,60)_ = 4.48, p < 0.01]. In comparison to E14 grafts, E12 grafts produced a higher yield of both types of dopamine neurons in the dSTR [p < 0.001] and N.Acc [p < 0.05] and a higher yield of A10-like neurons in the HPC [p < 0.05]. This effect of the donor age group was not unexpected since E12 grafts yielded a significantly higher number of TH-ir cells than E14 grafts in all transplantation sites apart from the PFC. The number of Girk2-ir/TH-ir cells was significantly higher than Calbindin-ir/TH-ir cells in grafts in the dSTR [p < 0.001] and in the N.Acc [p < 0.001] in both donor age groups and lower in the HPC in E12 grafts [p < 0.05]. Most importantly, E12 VM grafts produced the highest yield of A9-like neurons in the dSTR than anywhere else [Fig. 5I; p < 0.001], with the yield in grafts in N.Acc coming second and being significantly higher than that found in grafts in the PFC [Fig. 5I; p < 0.05] and HPC [Fig. 5I; p < 0.001]. In the E14 donor age group, grafts in the dSTR and N.Acc contained a comparable number of A9-like neurons that was higher than that in grafts in the PFC and HPC [Fig. 5I; both p < 0.05]. The yield of A10-like dopamine neurons in E14 grafts in N.Acc was higher than in other brain regions but this did not reach statistical significance [Fig. 5J; simple effect of Transplantation Site for E14 group, F_(3,60)_ = 0.85, n.s.]. In the E12 donor age group [Fig. 5J; simple effect of Transplantation Site for E12 group, F_(3,60)_ = 3.5, p < 0.05], only the number of A10-like neurons in grafts in the dSTR was significantly higher than in grafts in the PFC [p < 0.05].

In order to account for the difference in TH-ir cell numbers between two donor age groups, the yield of TH-ir neurons that were double-labelled with Girk2 and with Calbindin was calculated as a percentage of overall number of dopamine neurons. The percentages of Girk2-ir/TH-ir and Calbindin-ir/TH-ir cells in the dSTR and N.Acc added up to 105%, confirming an overlap in Girk2 and Calbindin stained cells. Due to limited availability of tissue and technical challenges, TH, Girk2 and Calbindin triple-staining was not undertaken. However, we were able to estimate an overlap of 11–17% in Girk2 and Calbindin staining in grafted rat VM by double IHC in pilot experiments (Fjodorova, 2013). Interestingly, the sum of percentages of A9- and A10-like dopamine neurons was 78% and 69% in grafts in the PFC and HPC, respectively, indicating that almost a third of TH-ir neurons failed to co-express Girk2 or Calbindin. The influence of A9 but not A10 dopaminergic targets in the transplantation site on the differentiation of grafted dopamine progenitors persisted after accounting for differential survival of E12 and E14 VM-derived grafts (Fig. 5K,L). Indeed, there was a highly significant difference between the percentages of A9- and A10-like neurons in the grafts [within-subject factor – Staining, F_(1,60)_ = 268.87, p < 0.001] influenced only by the host environment and not the donor age group [Transplantation Site, F_(3,60)_ = 91.81, p < 0.001; Donor Age, F_(1,60)_ = 1.14, n.s.; Transplantation Site × Donor Age, F_(3,60)_ = 1.82, n.s.]. The gain in the Girk2-ir/TH-ir and Calbindin-ir/TH-ir cell yields in the E12 donor age group did not outweigh the general increase in the number of TH-ir neurons between the two donor age groups. Significant differences between the percentages of Girk2-ir/TH-ir and Calbindin-ir/TH-ir neurons (Fig. 5K,L) corresponded to significant differences between the numbers of A9- and A10-like neurons in different graft groups (Fig. 5I,J). A higher percentage of Girk2-ir/TH-ir cells was observed in grafts in the dSTR than N.Acc and it continued to decrease in grafts in the PFC and HPC (Fig. 5K). Thus, although the total number of TH-ir neurons was affected by the environment, the decrease in Girk2-ir/TH-ir cell numbers outweighed the general decrease. There was a small increase in the percentage of Calbindin-ir/TH-ir cells in grafts in the N.Acc as compared to grafts in other transplantation sites in both donor age groups (Fig. 5L). However, even though there was a main effect of the transplantation site on the percentage of Calbindin-ir/TH-ir cells [Transplantation Site, F_(3,60)_ = 4.68, p < 0.01] no significant differences between individual groups were found.

### 3.5 Transplantation site influences distribution of A9- and A10-like dopamine neurons within ventral mesencephalic grafts

**Fig. 6:**
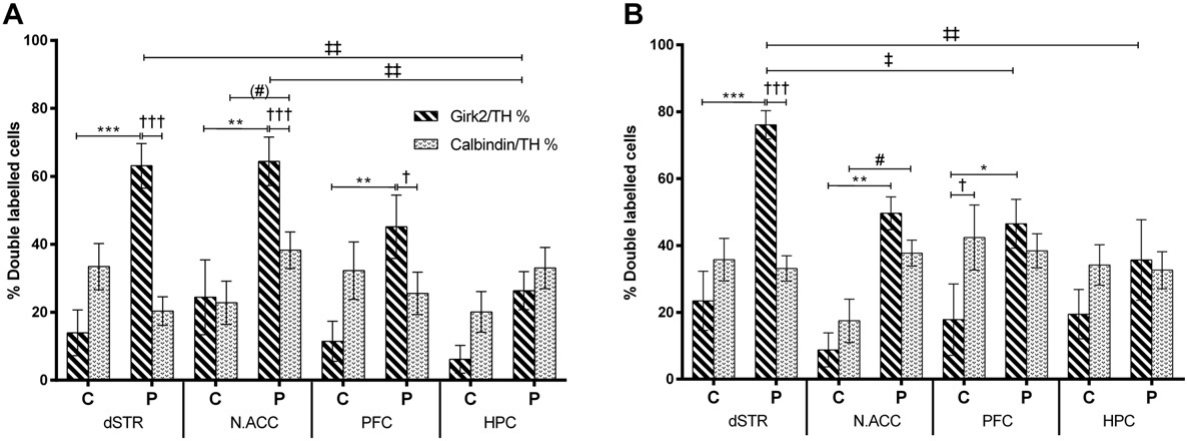
Transplantation site influences distribution of A9- and A10-like dopamine neurons within VM grafts. Percentages of Girk2-ir/TH-ir and Calbindin-ir/TH-ir neurons in the periphery and in the centre of E12 (A) and E14 grafts (B) at 6 weeks post-transplantation. Note the decrease in the percentage of Girk2-ir/TH-ir neurons in the periphery of grafts in HPC compared to grafts in other brain regions. The presence of A10 dopamine innervation of the N.Acc significantly increased the percentage of A10-like neurons in the periphery of the graft compared to the graft core in E14 group. Columns depict group means; error bars illustrate ± SEM; significance levels: *p < 0.05, **p < 0.01, ***p < 0.001 and accordingly for other symbols; different symbols are used to depict significant differences between distinct groups as follows: *centre vs periphery for Girk2/TH %, #centre vs periphery for Calbindin/TH % [special case (#) p < 0.1], †differences between Girk2/TH % and Calbindin/TH %, ‡differences between transplantation sites. Abbreviations: centre (C), periphery (P), dorsal striatum (dSTR), nucleus accumbens (N.Acc), prefrontal cortex (PFC), hippocampus (HPC).

Graft morphology was inspected to determine the distribution of dopamine neurons in the graft, with particular reference to their position either in the periphery or in the central core of the graft. Analysis showed that the majority of TH-ir neurons in the periphery were of the A9-like phenotype in all grafts apart from grafts in HPC, while fewer TH-ir neurons co-expressed Girk2 than Calbindin in the centre of the grafts in most transplantation sites (Fig. 6). Girk2-ir/TH-ir neurons were found predominantly in the periphery of grafts rather than in the core while Calbindin-ir/TH-ir neurons were distributed more homogeneously within the graft. The populations of cells in the periphery were generally very different from those in the core [within-subject factor – Location, F_(1,60)_ = 47.29, p < 0.001]. The A9-like dopamine cells were affected more than the A10-like neurons by the location within the graft [Location × Staining, F_(1,60)_ = 55.04, p < 0.001; A9-like – p < 0.001; A10-like – n.s.]. Transplantation site also had an effect on the distribution of dopamine neuron populations within the grafts [Transplantation Site, F_(3,60)_ = 3.44, p < 0.05] while VM age did not [Donor Age, F_(1,60)_ = 2.67, n.s.]. In both donor age groups, A9-like and A10-like dopamine neurons were found mostly in the periphery of grafts in N.Acc but in grafts in the dSTR this was true for A9-like neurons only. There was a significantly higher percentage of A10-like neurons in the periphery than in the centre in grafts in N.Acc in E14 donor age group [Fig. 6B; p < 0.05] whereas this approached but did not reach statistical significance in E12 grafts [Fig. 6A; p < 0.1, n.s.]. Interestingly, percentages of Girk2-ir/TH-ir and Calbindin-ir/TH-ir cells in the periphery of grafts added up to 90–100% in all groups apart from the HPC group. There were ≈ 50% of TH-ir cells in the centre of the graft in all groups that did not co-express neither Girk2 nor Calbindin suggesting that they most likely silenced their A9- and A10-like phenotypes.

The percentage of A9-like dopamine neurons in the periphery of grafts was significantly lower in grafts in the HPC than in the dSTR [p < 0.01] and N.Acc [p < 0.01] in the E12 donor age group (Fig. 6A). In E14 grafts, the percentage of Girk2-ir/TH-ir cells was significantly lower in the periphery of grafts in the PFC [p < 0.05] and HPC [p < 0.01] than in the dSTR (Fig. 6B). There was a higher percentage of Calbindin-ir/TH-ir neurons in the periphery of E12 grafts in the N.Acc than in other transplantation sites but the difference was not significant. Similarly, a higher percentage of A10-like dopamine neurons was found in the periphery of E14 grafts in N.Acc and PFC than in the dSTR or HPC but the differences failed to reach statistical significance.

## 4 Discussion

Improvement of functional efficacy of the cell replacement therapy for PD is still a major challenge that needs to be overcome. It is of paramount importance to understand the underlying mechanisms that lead to superior survival and integration of transplanted dopamine neurons. Here we describe the importance of two critical factors, the presence of A9/A10 dopamine innervation targets in the transplantation site and the age/developmental stage of the donor tissue, in improving the yield of functionally important A9-like dopamine neuron component in rat VM grafts in a 6-OHDA lesion model of PD.

Several previous studies have highlighted the impact of donor age on the cell therapy outcomes, reporting that grafting of younger mouse and rat VM cells produces transplants with an enhanced dopamine neuron yield and a greater proportion of A9-like dopamine neurons, as a result of increased progenitor proliferation and survival and the presence of meningeal cells in tissue preparation at the time of grafting (Bye et al., 2012, Somaa et al., 2015, Thompson et al., 2005, Torres et al., 2008, Torres et al., 2007). Also, grafting of VMs into both dSTR and N.Acc and re-innervation of the entire host striatum and the overlying cerebral cortex has been described in detail in the past (Nikkhah et al., 1994a, Nikkhah et al., 1994b). However, the extent to which commitment of VM progenitors at different stages in development to a specific dopamine neuron phenotype can be influenced by the host environment surrounding the graft has not previously been resolved.

The degree of innervation, patterning of regenerating axons and sprouting of appropriate transplanted neurons into the target have all been shown to be influenced by the host environment in studies in the dSTR (Thompson et al., 2005), HPC (Björklund and Stenevi, 1981) and sympathetic ganglia (Olson and Malmfors, 1970). This neuron-target interaction has been investigated and previous research has demonstrated that when transplanted in the vicinity of the dopamine-depleted dSTR, e.g. into the adjacent cortex or ventricle, grafts derived from human foetal VM tissue exhibited a target specific TH-ir fibre outgrowth (Stromberg et al., 1992). Also, dopamine neurons from the VM after transplantation to the HPC ramified extensively in the denervated perforant path zone but showed no tendency to grow into the normal terminal zones of the noradrenergic afferents (Björklund et al., 1976). Significant differences in the A9- and A10-like dopamine neuron populations within striatal grafts observed in mouse and patient studies (Mendez et al., 2005, Thompson et al., 2005) suggest that the host striatum may preferentially influence the development and/or survival of A9-like neurons. We therefore sought perform a systematic comparison and examine the effect that the A9 and/or A10 dopaminergic target status of the transplantation site may have on midbrain dopamine neuron differentiation and survival in younger (E12) and conventional (E14) VM grafts.

Within the present study each animal received two VM grafts, the first transplant into either dSTR or N.Acc and the second into either PFC or HPC. Grafts in either the PFC or HPC did not interfere with drug-induced rotational response provided by grafts in either dSTR or N.Acc. The behavioural analysis of drug-induced rotation test revealed a significant recovery of rotational bias in rats grafted with VM tissue into the dSTR in both donor age groups (Fig. 1A). This is in line with previous observations of rat dopaminergic grafts providing sufficient dopamine supply to the dSTR to counteract severe rotational deficits induced by unilateral nigrostriatal pathway loss (Grealish et al., 2010, Torres et al., 2008). Conversely, grafts in the N.Acc exacerbated the rotational bias toward the lesioned side in response to the drug indicating that dopamine released by the graft had a net excitatory effect on N.Acc neurons (Fig. 1B), compatible with the dual process model of rotation first proposed by Kelly and Moore (1976). Indeed, dopamine has been shown to excite N.Acc neurons by preferentially inhibiting GABA release during sustained stimulation in rat brain slices and inactivating dopamine neurons reduces excitation of N.Acc neurons in rats (Hjelmstad, 2004, Yun et al., 2004). However, grafts in the E12 N.Acc group had no effect on the rotational bias in response to amphetamine most likely due to a higher degree of graft-derived innervation in the E12 group and dopamine fibres reaching the adjacent dSTR and modulating dopamine release in N.Acc (Fig. 3B,F). Taken together these findings confirm that dopaminergic grafts survived and functionally integrated into the host tissue providing regulated dopamine release in a physiological manner.

The histological findings, including the total numbers of surviving transplanted NeuN-ir neurons (Fig. 2E) and TH-ir neurons (Fig. 4A), showed that the transplantation site had little influence on the overall survival of VM grafts in either donor age group. As previously, the use of younger donor age tissue was seen to be beneficial to dopaminergic neuron survival. When compared with 35,000 dopamine neurons in the healthy adult rat VM (German and Manaye, 1993), current TH-ir neuron yield in grafts in the E12 group, derived from implantation of 1/2 VM, represents up to 28% of the expected adult complement, a much better result than conventional E14 grafts, which were able to achieve only 12%. Of relevance, a recent study by Torres et al. (2007) reported survival rates of dopamine neurons of over 35% when using younger (E12) embryos. A similar impact of the donor age was observed in mouse VM grafts where DA neuron yield was 2.6-fold higher in grafts derived from the E10 VM tissue as compared to the E12 VM tissue (Bye et al., 2012). In another study, multiple rat E12 VM-derived intrastriatal grafts produced an even higher yield of DA neurons (75% survival rate) and innervated a greater volume of the dSTR (Torres et al., 2008). Similarly, in the current experiment, dopaminergic innervation arising from E12 VM grafts appeared denser and more extensive in all cerebral targets compared to E14 VM grafts (Fig. 3). Additionally, E12 grafts were bigger and displayed higher density of TH-ir neurons than E14 grafts in most locations (Fig. 4C,D). Thus, the increase in the total number of TH-ir cells in grafts derived from younger donor tissues can be attributed to a denser distribution of dopamine neurons within the graft which is accompanied by a slight increase in the graft volume.

As in the adult midbrain, VM grafts in this study contained two recognisable classes of dopamine neurons: large elongated Girk2-ir A9-like dopamine neurons of SNpc and smaller round Calbindin-ir A10-like dopamine neurons of the VTA (Fig. 5). We acknowledge that there is a 10–25% overlap in Girk2 and Calbindin expression in midbrain dopaminergic neurons (Bye et al., 2012, Grealish et al., 2010, Reyes et al., 2012). However, these two markers together with cellular morphology are currently the best two tools available to distinguish between A9- and A10-like neurons in the grafts. Interestingly, we observed both A9- and A10-like dopamine neurons in abundance in grafts in all transplantation sites. Most importantly, the yield of A9-like neurons followed a pattern with the highest number of Girk2-ir/TH-ir cells found in grafts in the dSTR, which contains targets of A9 innervation from the SNpc, followed by a progressive decrease of this neuron population in E12 grafts in the N.Acc, PFC and HPC in the absence of the A9 dopamine innervation targets (Fig. 5I,K). E14 grafts in the dSTR and N.Acc yielded similar numbers of the Girk2-ir/TH-ir cells indicating that the differentiation/maturation of developmentally older dopamine cells was less affected by the presence of environmental cues compared to younger midbrain progenitors. Interestingly, it has been reported that responsiveness to cues to direct phenotypic differentiation is downregulated in older neurons (Van den Heuvel and Pasterkamp, 2008), which is the case observed in the present study. Nevertheless, in the absence of A9 targets for innervation, E14 grafts in the PFC and HPC produced significantly lower numbers of A9-like neurons than grafts in the dSTR and N.Acc. The environment rich in A10 innervation targets in the N.Acc and PFC did not promote a significant increase in the Calbindin-ir/TH-ir neuron population compared to grafts in the dSTR or HPC (Fig. 5J,L). Although the trend for changes in the population of A10-like neurons went in the hypothesised direction for E14 VM grafts, it did not reach statistical significance. These observations are in agreement with a recent study that utilised mouse-derived VM grafts to investigate environmental cues provided by meningeal cells in the developing midbrain and reported that older VM tissue as well as A10-like neuron population were less affected (Somaa et al., 2015).

Consistent with previous reports, A9-like neurons were predominantly located in the periphery of grafts while A10-like neurons were more homogeneously distributed within the graft (Mendez et al., 2005, Mendez et al., 2008, Thompson et al., 2005). The effect of the environment on two dopamine neuron subtypes was more apparent in the periphery of grafts rather than in the centre probably because neurons there were more exposed to the surrounding cues, or because neurons in the core were unable to extend axons in to the target area. Indeed, around half of TH-ir neurons in the core of the grafts failed to co-express either Girk2 or Calbindin and acquire a mature dopamine neuron phenotype, a phenomenon also observed in hVM grafts in PD patients (Mendez et al., 2005, Mendez et al., 2008). Most interestingly, striatal environment was found to positively influence the yield of A9-like neurons in the periphery of grafts as compared to transplants in the PFC and HPC in both donor age groups (Fig. 6). Without the necessary environmental cues considerably fewer dopamine precursor cells matured into functional A9-like neurons in the periphery of grafts implanted in the PFC and HPC. Furthermore, grafts in the N.Acc contained slightly more A10-like neurons in the periphery than grafts in other locations, but the differences were not significant. Nevertheless, Calbindin-ir/TH-ir neurons were found predominantly in the periphery of grafts in N.Acc rather than in the centre, in both donor age groups, indicating that environmental cues in the N.Acc might have influenced differentiation and/or survival of A10-like dopamine progenitors or attracted their migration to the periphery of the graft.

The absence of a greater effect of the transplantation site on the A10-like neuron population in the grafts could be attributed, at least in part, to the fact that a unilateral 6-OHDA lesion of the medial forebrain bundle causes only a partial loss of dopamine neurons in the VTA and subsequently achieves only a partial denervation of the N.Acc and PFC. Indeed, previous studies have suggested that although dopamine grafts survive equally well both in the intact and lesioned striatal environment (Dunnett et al., 1988, Schmidt et al., 1981), integration of the grafts in adult recipients was reportedly poor unless the host systems were sufficiently denervated. Taken together, the present results suggest that the level of dopamine depletion in either A9 (dSTR) or A10 (N.Acc) input nuclei in the basal ganglia may influence the preferential yield of dopamine neuron phenotypes in the grafts.

A further major determinant of a successful cell therapy is to achieve adequate innervation of target tissue by the graft and restoration of dopamine supply. Irrespective of the dopamine neuron yield in a transplant, failure to repair functional connections in the dSTR does not improve motor function (Fricker-Gates and Dunnett, 2002). Meningeal cells overlying the VM have been recently implicated not only in superior dopamine neuron yield in the graft but, most importantly, also in an increased innervation of the host brain (Somaa et al., 2015). Those authors demonstrated that an increase in innervation density is enhanced by stromal derived factor-1 (SDF1) secreted by meningeal cells in the grafts. Current and previous observations of a greater fibre outgrowth achieved by grafts derived from younger VM tissue may be similarly linked to the influence of meningeal cells as they were not removed during graft preparations from rat E12 or mouse E10 VM tissue. In contrast, meningeal cells were not included in older VM-derived grafts (Bye et al., 2012, Torres et al., 2008). Previous research has demonstrated that restoration of finer motor skills requires a more global restoration of the basal ganglia circuitry which can be achieved by simultaneous grafts in the dSTR, SNpc and the subthalamic nucleus (Dunnett et al., 1989, Mukhida et al., 2001, Mukhida et al., 2008). In clinical trials, PD patients receive transplants primarily into the putamen and in some cases also into either the caudate nucleus or SNpc.

Taken together, our current findings not only expand our understanding of grafted dopamine neuron differentiation and maturation in the host brain, but also have significant implications for further improving dopamine cell therapy in PD in the future. Replacement of lost neurons can be targeted to individual needs of each patient taking into account specific patterns of neurodegeneration, thereby allowing more precise restoration of the circuitry and neurotransmitter release. In the present study, we demonstrated the ability of younger VM tissue to use environmental cues in the transplantation site to guide terminal A9- and A10-like phenotypic differentiation of dopamine neuron progenitors in the graft. Thus, by transplanting younger human VM tissue into different sites in the caudate nucleus, putamen and SNpc, it may be possible to enrich grafts for a specific dopamine neuron population to achieve a more extensive restoration of circuitry and improve the quality of life for patients.

## Author contributions

MF, EMT, and SBD designed the experiment. MF and EMT ran the experiment and analysed the data. MF, EMT, and SBD wrote the manuscript.

## Potential conflicts of interest

Drs. Fjodorova and Torres, and Professor Dunnett reported no biomedical financial interests or potential conflicts of interest.

## Figures and Tables

**Table 1:** Group allocation for transplantation. Animals were distributed in 8 balanced groups based on the number of amphetamine-induced net ipsilateral rotations following 6-OHDA lesion. Data are presented as group means ± SEM, (number of animals per group [surviving primary grafts, dSTR or N.Acc/surviving secondary grafts, PFC or HPC]).

Donor age	Transplantation site			
	dSTR PFC	N.Acc PFC	dSTR HPC	N.Acc HPC
E12	1242 ± 273 (n = 5 [5/5])	1268 ± 260 (n = 4 [4/3])	1307 ± 500 (n = 4 [4/4])	1245 ± 264 (n = 5 [5/5])
E14	1190 ± 168 (n = 5 [6/5])	1388 ± 380 (n = 4 [3/2])	1298 ± 428 (n = 4 [4/4])	1245 ± 227 (n = 5 [5/4])
